# Feed restriction and a diet's caloric value: The influence on the aerobic and anaerobic capacity of rats

**DOI:** 10.1186/1550-2783-9-10

**Published:** 2012-03-26

**Authors:** Leandro Pereira de Moura, Carlos Augusto Kalva-Filho, João Paulo Loures, Maria de Sousa Silva, Lucas Pilla Zorzetto, Marcelo Costa Junior, Michel Barbosa de Araújo, Rodrigo Augusto Dalia, Maria Alice Rostom de Mello

**Affiliations:** 1 Laboratory of Nutrition, Metabolism and Exercise, Department of Physical Education, Universidade Estadual Paulista (UNESP), 24ª avenue n° 1515, P.O. Box 199, Bela Vista, Rio Claro, SP, Brazil; 2 Laboratory of Exercise Physiology (LAFE), Department of Physical Education, Universidade Estadual Paulista (UNESP), Roberto Simonsen Street, 305, Presidente Prudente, SP, Brazil; 3 Education, Universidade Estadual Paulista (UNESP), Avenida 24ª n° 1515, P.O. Box 199 Bela Vista, Rio Claro 13506-900, SP, Brazil

**Keywords:** Diet, Feed restriction, Lactate minimum

## Abstract

**Background:**

The influence of feed restriction and different diet's caloric value on the aerobic and anaerobic capacity is unclear in the literature. Thus, the objectives of this study were to determine the possible influences of two diets with different caloric values and the influence of feed restriction on the aerobic (anaerobic threshold: AT) and anaerobic (time to exhaustion: Tlim) variables measured by a lactate minimum test (LM) in rats.

**Methods:**

We used 40 adult Wistar rats. The animals were divided into four groups: *ad libitum *commercial Purina^® ^diet (3028.0 Kcal/kg) (ALP), restricted commercial Purina^® ^diet (RAP), *ad libitum *semi-purified AIN-93 diet (3802.7 Kcal/kg) (ALD) and restricted semi-purified AIN-93 diet (RAD). The animals performed LM at the end of the experiment, 48 h before euthanasia. Comparisons between groups were performed by analysis of variance (p < 0,05).

**Results:**

At the end of the experiment, the weights of the rats in the groups with the restricted diets were significantly lower than those in the groups with *ad libitum *diet intakes. In addition, the ALD group had higher amounts of adipose tissue. With respect to energetic substrates, the groups subjected to diet restriction had significantly higher levels of liver and muscle glycogen. There were no differences between the groups with respect to AT; however, the ALD group had lower lactatemia at the AT intensity and higher Tlim than the other groups.

**Conclusions:**

We conclude that dietary restriction induces changes in energetic substrates and that *ad libitum *intake of a semi-purified AIN-93 diet results in an increase in adipose tissue, likely reducing the density of the animals in water and favouring their performance during the swimming exercises.

## Background

Several authors have studied the effects of caloric restriction on body composition and metabolic variables in both humans [1-3] and animals [4]. Reducing daily feed intake to 20 to 40% below *ad libitum *levels, or providing feed intermittently rather than continuously, has been found to significantly reduce the risk of chronic degenerative diseases such as cancer, type-II diabetes and kidney diseases, and to prolong the life span of laboratory rats and mice by 40% without causing malnutrition [4-7].

However, excessive dietary restriction can lead to malnutrition and physiological changes that lead to decreases in sympathetic nervous system activity, changes in thyroid metabolism, reductions in insulin concentrations and changes in glucagon, growth hormone and glucocorticoid secretion [8]. Furthermore, these changes may promote the mobilisation of endogenous substrates, leading to increased circulation of fatty acids and increased protein catabolism (including a reduction in muscle protein - [9]), reflecting the decrease in energy expenditures [8].

According to Vanittalie and Yang [10], additional changes may occur to the protein content of heart muscle fibres. Individuals who have lost a significant amount of weight (30% of initial weight) have reduced cardiac mass, and heart muscle fibre atrophy occurs when dietary restriction is implemented in excess, thus reducing the vital capacity of individuals and potentially impairing aerobic and anaerobic performance. These changes, which occur because of an energy deficit, may lead to vital changes in the body.

Given the limitations on human research, animal models have become very important tools for studying many areas of science, including exercise physiology. The use of overweight and inactive animals as controls can affect the results of studies. Therefore, researchers are currently proposing that new procedures for maintaining laboratory animals be evaluated and implemented in order to assure that the control animals are fed in portions rather than *ad libitum*, and that they have adequate environmental stimulation and physical activity [11].

The use of a "control diet" is fundamentally important to researchers. Two types of "control diets" are frequently used in physiological studies: a commercial rodent diet (Purina^®^) and a diet proposed by the American Institute of Nutrition in 1993 (AIN-93) [12]. However, these two diets have different caloric contents (Purina^®^: 3028.0 Kcal/kg and American Institute of Nutrition diet (AIN-93 M): 3802.7 Kcal/kg) that are not usually considered. Moreover, it is important to determine the possible effects of the feeding protocol and the different diets fed to rodents that are used in exercise physiology studies.

The lactate minimum test has been used in exercise physiology studies of both humans [13-16] and rodents [17-19] because it enables researchers to determine aerobic and anaerobic capabilities in a single test [20,21]. However, the possible effects of administering diets with different caloric values and feed restriction on the parameters provided by the lactate minimum test are not well understood in the literature. Therefore, the purpose of this study was to evaluate the effects of dietary restriction (60% of *ad libitum *intake) of two control diets (commercial Purina^® ^and American Institute of Nutrition diet (AIN-93 M)) on the aerobic and anaerobic capacity of Wistar rats, as determined by the lactate minimum test.

## Methods

### Animals and animal care

The duration of study was one month and we used 40 Wistar rats that were from 90 days old at the beginning of the experiment and had body weights of 406.9 ± 39.44 g. The animals were housed in polyethylene cages measuring 37 × 31 × 16 cm (five rats per cage) at room temperature (25°C) with a 12-hour light/dark photoperiod. All procedures involving animals were submitted to and approved by the Ethics Committee on Animal Use in Research of the Biosciences Institute of UNESP, Rio Claro Campus (protocol number: 2011/6274).

The animals were divided into four groups with 10 animals per group, depending on the diet and mode of administration. Two groups had access to commercial feed (Purina^®^): one *ad libitum *(ALP) and the other restricted (RAP). The two other groups had access to the diet proposed by the American Institute of Nutrition in 1993 (AIN-93 M): one *ad libitum *(ALD) and the other restricted (RAD). Feed intake for the animals in the *ad libitum *groups was recorded daily. Thus, for the animals on feed restriction, feed was offered in an amount corresponding to 60% of the average amount consumed by the *ad libitum *groups the previous day. This protocol was selected to allow for dietary restriction without causing malnutrition [4]. All groups had free access to water.

### Diet compositions

Commercial Purina^® ^Diet (Paulínia/SP, Brazil)

This diet was composed of 43.7% carbohydrates, 23% protein, and 4% fat at 3,028 kcal/g. The remainder of the ingredients were comprised of minerals, fibre, and vitamins.

*AIN-93M (Semi-purified diet, according to the American Institute of Nutrition, AIN-93M*; [12])

The diet was composed of 70% carbohydrates, 14% protein, and 4% fat at 3,802.7 kcal/g. The remainder of the ingredients were comprised of minerals, fibre, and vitamins.

### Adaptation to water

Before undergoing the lactate minimum protocol, all the animals were adapted only one time to water. The adaptation occurred over a total period of five continuous days, by placing the animals in shallow water in the tank where the tests occurred. The water temperature was maintained at 31 ± 1°C [19]. The purpose of the adaptation was to reduce the stress of the animals, without promoting physiological adaptations that result from physical training.

### Evaluation of aerobic and anaerobic capacity

To determine acutely aerobic and anaerobic capacity, we used the lactate minimum test, which enabled us to determine both parameters in a single protocol [20,21]. This test consists of an induction phase to hyperlactatemia (anaerobic exercise) followed by progressive exercise.

The induction phase consisted of two efforts with a load equivalent to 13% of the animals' body weight. The first effort lasted 30 s, followed by a 30-s passive recovery period. After the recovery period, the animals performed a maximum effort to obtain the time to exhaustion, considered as the parameter of anaerobic fitness. Nine minutes after the exhaustion period, we collected 25 μl of blood via a cut at the distal end of the tail to determine lactate concentrations.

After collecting the blood, the animals began a progressive phase with an initial intensity of 4.0% of body weight, which was increased by increments of 0.5% of body weight over 5 min intervals. At the end of each stage, 25 μl of blood was collected to determine lactate concentrations. The anaerobic threshold, considered as the parameter for aerobic capacity, was equivalent to the zero derivative of a second-order polynomial fit that was obtained from the relationship between lactate concentrations and the exercise intensity. Consequently, we determined lactate concentrations based on the anaerobic threshold.

During all the efforts, the animals were placed individually in tanks (100 × 80 × 80 cm) containing water at 31 ± 1°C. Blood samples were collected using calibrated capillary tubes and heparinised, and blood lactate was determined using an enzymatic method [22].

### Evaluations conducted during the intervention and before euthanasia

Throughout the experimental period, the body weights (all groups) and feed intakes *(ad libitum *group) were recorded daily using an analytical balance. The results were analysed based on the weight change of the animals (weight change = initial weight - final weight).

### Parameters obtained following euthanasia

At the end of the experiment, animals were anesthetised in a CO_2 _chamber, 48 h after measuring the lactate minimum test. Blood was collected directly from the heart via cardiac puncture with disposable needles and syringes. The blood was subsequently centrifuged at 3000 rpm for 15 m, and the serum supernatant was used to determine glucose, total protein and albumin content [23] using commercially available colorimetric enzymatic kits (Labor-lab, Brazil).

Samples of the gastrocnemius (red and white portions) and soleus muscles were collected and used to assess glycogen [24] and triglyceride content [23]. We also collected liver samples for glycogen [24] and total lipid analyses [23]. All the samples were homogenised in a Polytron^® ^for 20 s at maximum speed. They were then centrifuged at 10,000 rpm for 5 min at 4°C prior to the analyses.

### Statistical analysis

The normality of the data was confirmed using the *Shapiro-Wilk *test. The results are presented as the mean ± standard deviation. Comparisons between groups were performed by analysis of variance (one-way ANOVA) and the Newman-Keuls Post-hoc test when necessary. For all the analyses, the level of significance was set at *p *< 0.05 (Statistica 7; Statsoft, USA).

## Results

**Figure 1:**
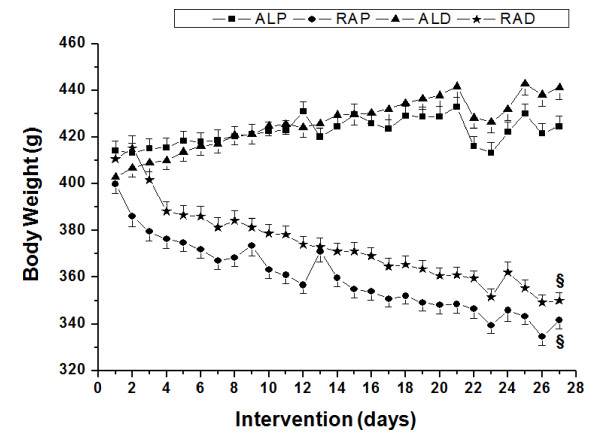
**Daily values of body weight for animals in the *ad libitum *commercial diet (ALP), restricted commercial diet (RAP), *ad libitum *AIN-93 diet (ALD) and restricted AIN-93 diet (RAD) groups**. § Significant difference compared to the *ad libitum *groups (p < 0.05).

During the interventions in this study, the animals from the RAP and RAD groups showed a significant decrease in body weight over the course of the experimental period (Figure 1). However, neither group showed any clinical indications of malnutrition, such as hypoalbuminemia, hypoproteinemia or high lipid content in the liver (LIP_LIV_).

**Figure 2:**
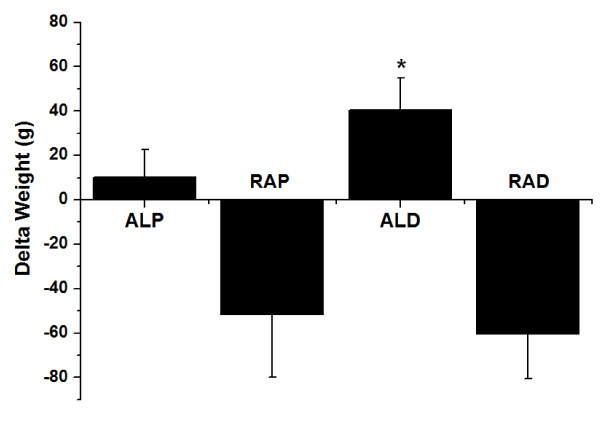
**Change in body weight (initial weight - final weight) between the *ad libitum *commercial diet (ALP), restricted commercial diet (RAP), *ad libitum *AIN-93 diet (ALD) and restricted AIN-93 diet (RAD)**. * Significant difference compared to the ALP group (p < 0.05).

Nevertheless, animals in the RAD group had significantly lower LIP_LIV _compared to the ALP and ALD groups (p < 0.05) (Table 1). The change in weight during the intervention (weight change = initial weight - final weight) was significantly higher for the ALD group compared to the ALP group (Figure 2). Furthermore, the ALD group had greater amounts of subcutaneous adipose tissue (p < 0.05) than the other groups. In contrast, the RAP and RAD groups had significantly less adipose tissue in the mesenteric and retroperitoneal regions compared to the *ad libitum *groups (Table 2).

**Table 1:** Concentrations of albumin, total protein and liver lipids observed in the *ad libitum *and restricted groups

	ALP	RAP	ALD	RAD
ALB	2.8 ± 0.4	2.8 ± 0.1	2.9 ± 0.2	2.9 ± 0.1
PRO_TOTAL_	6.8 ± 0.6	4.2 ± 0.5	4.8 ± 1.3	3.6 ± 0.4
LIP_LIV_	4.6 ± 0.6	4.2 ± 0.5	4.8 ± 1.2	3.6 ± 0.4 *°

*ALP Ad libitum *commercial (Purina^®^) diet group, *RAP *Restricted commercial (Purina^®^) diet group, *ALD Ad libitum *semi-purified AIN-93 diet group, *RAD *Restricted semi-purified AIN-93 diet group, *ALB *Concentrations of albumin (g/dL), *PRO_TOTAL _*Total protein (g/dL), *LIP_LIV _*Liver lipids (mg/100 mg); * Significant difference compared to the ALP group (p < 0.05); °significant difference compared to the ALD group (p < 0.05)

**Table 2:** Adipose tissue weight both *ad libitum *commercial and AIN-93 groups and their respective feed restricted groups

	ALP	RAP	ALD	RAD
SUB	1.6 ± 0.8	1.1 ± 0.4	2.2 ± 0.4 ‡	1.0 ± 0.4
MESE	2.6 ± 1.4	1.1 ± 0.7 *°	2.8 ± 1.0	1.7 ± 0.7 *°
RETRO	3.0 ± 2.0	1.3 ± 1.0 *°	3.5 ± 1.4	1.6 ± 0.7 *°

*ALP Ad libitum *commercial (Purina^®^) diet group, *RAP *Restricted commercial (Purina^®^) diet group, *ALD Ad libitum *semi-purified AIN-93 diet group, *RAD *Restricted semi-purified AIN-93 diet group, *SUB *from subcutaneous tissue(g), *MESE *mesenteric tissue (g), *RETRO *retroperitoneal tissues (g); ‡ Significant difference compared to all groups * Significant difference compared to the ALP group (p < 0.05); °significant difference compared to the ALD group (p < 0.05)

The levels of liver glycogen (GLYC_LIV_) in the RAD and RAP groups were significantly higher (p < 0.05) than those found in the ALP and ALD groups. Moreover, the quantities of soleus muscle glycogen (GLYC_SOL_) in the RAP group were also higher than in the ALP and ALD groups (p < 0.05). There were no significant differences between the groups with respect to the levels of triglycerides found in the soleus (TG_SOL_) and gastrocnemius (TG_GAS_) muscles (Table 3).

**Table 3:** Values of the levels of liver glycogen and soleus (GLYC_SOL_; mg/100 mg) and gastrocnemius muscles, and the levels of triglyceride from this tissues

	ALP	RAP	ALD	RAD
GLYC_SOL_	0.2 ± 0.1	0.4 ± 0.1 *°	0.2 ± 0.1	0.3 ± 0.1 *
GLYCO_GAS_	0.1 ± 0.03	0.3 ± 0.1 *	0.2 ± 0.1	0.2 ± 0.1
GLYC_LIV_	0.9 ± 0.2	3.9° ± 1 *	0.8 ± 0.1	3.7 ± 0.5 *°
TG_SOL_	0.3 ± 0.2	0.2 ± 0.1	0.2 ± 0.1	0.3 ± 0.2
TG_GAS_	0.2 ± 0.1	0.2 ± 0.1	0.2 ± 0.1	0.3 ± 0.2

*ALP Ad libitum *commercial (Purina^®^) diet group, *RAP *Restricted commercial (Purina^®^) diet group, *ALD Ad libitum *semi-purified AIN-93 diet group, *RAD *Restricted semi-purified AIN-93 diet group, *GLYC_LIV _*glycogen content of liver (mg/100 mg), *GLYC_GAS _*glycogen content of gastrocnemius (mg/100 mg), *GLYC_SOL _*glycogen content of soleus (mg/100 mg), *TG_SOL _*triglyceride content of soleus (mg/100 mg), *TG_GAS _*triglyceride content of gastrocnemius (mg/100 mg) * Significant difference compared to the ALP group (p < 0.05); °significant differences compared to the ALD group (p < 0.05)

Table 4 shows the values for aerobic capacity, lactate concentrations and anaerobic capacity (time to exhaustion) determined using the lactate minimum test in all the groups studied. The anaerobic threshold values did not differ between the groups, whereas the lactate concentrations values were significantly lower (p < 0.05) in the ALD group compared to other groups. In addition, the ALD group had higher time to exhaustion (p < 0.05) compared to the ALP and RAP groups (Table 4).

**Table 4:** Aerobic and anaerobic capacity and the concentration of lactate corresponding to aerobic capacity

	ALP	RAP	ALD	RAD
AT (%PC)	4.4 ± 0.4	4.8 ± 0.9	4.7 ± 0.3	4.3 ± 0.3
[Lac]_AT _(mM)	6.6 ± 1.1	7 ± 0.7	5.2 ± 1 ‡	6.7 ± 0.9
Tlim (s)	63.4 ± 18.2	72.10 ± 47	116.5 ± 26.3†	94.1 ± 50

*ALP Ad libitum *commercial (Purina^®^) diet group, *RAP *Restricted commercial (Purina^®^) diet group, *ALD Ad libitum *semi-purified AIN-93 diet group, *RAD *Restricted semi-purified AIN-93 diet group, *AT *aerobic capacity, *Tlim *anaerobic capacity, *[Lac]_AT _*lactate concentration corresponding to aerobic capacity; † Significant difference compared to the ALP and RAP groups (p < 0.05); ‡ Significant difference compared to all groups (p < 0.05)

## Discussion

The principle findings of this study demonstrate that a 40% restriction on the amount of feed offered to the rats did not cause malnutrition in adult Wistar rats over a four-week period. In addition, the caloric difference between the two control diets used (Purina^®^: 3028.0 Kcal/kg and AIN-93 M: 3802.7 Kcal/kg) did not cause changes in the levels of muscle and liver glycogen, whereas the way in which the diets were administered resulted in increased levels of these substrates in the animals in the RAP and RAD groups. Additionally, the American Institute of Nutrition diet (AIN-93 M) that was administered *ad libitum *improved the aerobic and anaerobic capacity of the ALD group, probably due to the lower density of these animals in water.

Malnutrition in animals is often characterised by low serum albumin and total protein concentrations and high levels of liver lipids [18,25]. In the present study, the animals that had restricted access to feed (RAP and RAD) did not show these characteristics, confirming previous research [4]. In addition, studies have shown that dietary restriction (80 to 60% of *ad libitum *intake) decreases the risk of chronic degenerative diseases such as cancer, type-2 diabetes and kidney disease, prolonging the life span of laboratory rats and mice by up to 40% without causing malnutrition [5-7].

Comparing the effects of a standard diet (Purina^®^) to those of a freely administered high calorie diet, Chun, Lee, Kim, et al. [26] showed that animals on a high calorie diet have higher levels of body fat. These findings are consistent with the present study, where the ALD group, which was fed a higher caloric diet American Institute of Nutrition diet (AIN-93 M), showed more weight gain than the ALP group. According to Silva, Marcondes and Mello [27], animals that are subjected to high-fat diets tend to accumulate more fat than control animals.

The RAP and RAD groups showed higher glycogen values, primarily in the soleus muscle and liver, than those fed *ad libitum*. Corroborating these findings, Pedrosa, Tirapegui, Rogero, et al. [28], when comparing sedentary and trained animals, both with and without feed restriction (25 and 50% of *ad libitum *intake), observed higher muscle and liver glycogen values in the animals in the restricted groups. In addition, Wetter, Gazdag, Dean, et al. [29] observed similar or higher muscle glycogen values in rats subjected to feed restriction (60% of consumed by the *ad libitum *group), as has been demonstrated by other studies [30,31]. Conversely, these authors found higher liver glycogen values in animals fed *ad libitum*, suggesting that the influence of dietary restriction on the content of this substrate is dependent on the tissue analysed. In this regard, further studies are needed to determine the changes caused by dietary restriction on the mechanisms of glycogen synthesis and utilisation in different tissues.

The differences in the levels of muscular glycogen could influence aerobic and anaerobic capacity in animals, as determined using the lactate minimum test. However, there were no significant differences in the anaerobic threshold values between the groups, demonstrating that the diets and their form of administration did not influence the aerobic capacity of the animals. In addition, the loads corresponding to the anaerobic threshold in relation to body weight (4.5%) are similar to those reported by previous studies that used eutrophic rats [18,32,33] ARAÚJO et al., 2007).

However, the animals in the ALD group showed lower lactate concentrations values_. _This finding, together with the lower quantities of glycogen in the *ad libitum *groups, is consistent with those reported by Voltarelli, Gobatto and Mello [33], who observed lower lactate concentrations values in glycogen-depleted animals when comparing anaerobic threshold determined using lactate minimum test in a group of fed animals and a group of animals subjected to glycogen depletion.

The animals in the ALD group showed the same characteristics observed in humans subjects during a lactate minimum test after glycogen depletion, i.e., the intensity corresponding to the minimum lactate concentration was not influenced by a reduction in glycogen stores; however, the lactate concentrations were significantly lower upon depletion [20]. Further, the lactate concentrations and time to exhaustion values may have been influenced by the density of the animals in the ALD group, since these animals had an increase in body weight and body fat.

Araújo, Araújo, Dangelo, et al. [34] demonstrated that the anaerobic threshold in obese animals, as determined using maximal steady state lactate levels, may be higher than that in well-nourished animals, attributing these findings to the lower density of these animals in an aquatic environment. Thus, in our study, the intensity at the same workload may have been underestimated for animals that have higher levels of fat [35], resulting in the lower lactate concentrations values and higher time to exhaustion values seen in the ALD group. Therefore, more studies are needed to normalise the variables related to the increased loads used in lactateminimum test as a function of the body density of the animals.

The results of this study suggest that the caloric differences between the two diets did not noticeably influence the levels of muscle and liver glycogen, whereas these levels could be influenced by the form in which the diets were administered. However, the higher levels of glycogen seen in the RAP and RAD groups did not influence the aerobic and anaerobic capacity as determined using the lactate minimum test. In addition, the lower lactate concentrations and higher time to exhaustion values seen for the ALD group may be explained by the lower density of the animals in this group. Thus, one limitation of this study was the lack of quantification of the density of the animals and the use of loads that did not consider this variable in water.

In summary, feed restriction induced changes in energetic substrates, and *ad libitum *intake of a semi-purified American Institute of Nutrition diet (AIN-93 M) resulted in increased adipose tissue, which likely reduced the density of the animals in water and favoured their performance in the swimming exercise.

## Conclusion

From the results of this study, we can conclude that: 1) the animals in the diet-restricted groups showed no manifestations of malnutrition, indicating that the amount of feed offered (60% of that consumed by the *ad libitum *group) was sufficient; 2) the caloric differences in the diets studied did not alter the levels of muscle and liver glycogen, whereas the form of administration *(ad libitum *or restricted) did modify the quantities of these substrates; 3) the differences in the levels of glycogen between the two groups had little influence on the aerobic and anaerobic capacity of the animals; and 4) the ALD group animals may have had a lower density in water, which might have influenced the lactate concentrations and time to exhaustion values observed in this group.

## Abbreviations

AT: Anaerobic Threshold; Tlim: Time to Exhaustion; LM: Lactate Minimum Test; ALP: *Ad libitum *commercial (Purina^®^) diet group; RAP: Restricted commercial (Purina^®^) diet group; AIN-93: Diet proposed by the American Institute of Nutrition in 1993; ALD: *Ad libitum *semi-purified AIN-93 diet group; RAD: Restricted semi-purified AIN-93 diet group.; GH: Growth hormone; [LAC]: Lactate concentrations; [LAC]_AT_: Lactate concentrations at Anaerobic Threshold intensity.; WC: Weight change; ALB: Concentrations of albumin; PRO_TOTAL_: Total protein; LIP_LIV_: Lipid content in the liver; GLYC_LIV_: Glycogen content in the liver; GLYC_SOL_: Soleus muscle glycogen content; TG_SOL_: Triglycerides content in the soleus muscle; TG_GAS_: Triglycerides content in the gastrocnemius muscle.

## Competing interests

The authors declare that they have no competing interests.

## Authors' contributions

All authors were responsible for the experimental design, data collection, statistical analysis and preparation of the manuscript. All authors worked read and approved the final manuscript.
